# Three Dimensional Methodology to Characterize Large Dendritic Equiaxed Grains in Industrial Steel Ingots

**DOI:** 10.3390/ma11061007

**Published:** 2018-06-13

**Authors:** Marvin Gennesson, Julien Zollinger, Dominique Daloz, Bernard Rouat, Joëlle Demurger, Hervé Combeau

**Affiliations:** 1 Laboratory of Excellence on Design of Alloy Metals for low-mAss Structures (DAMAS), Université de Lorraine, 57073 Metz, France; julien.zollinger@univ-lorraine.fr (J.Z.); dominique.daloz@univ-lorraine.fr (D.D.); bernard.rouat@univ-lorraine.fr (B.R.); herve.combeau@univ-lorraine.fr (H.C.); 2 Department of Metallurgy & Materials Science and Engineering, Institut Jean Lamour, 2 allée André Guinier Campus Artem, 54000 Nancy, France; 3 Ascométal CREAS, Avenue de France, BP 70045, 57301 Hagondange, France; joelle.demurger@ascometal.com

**Keywords:** industrial ingot, steel, dendritic grain size

## Abstract

The primary phase grain size is a key parameter to understand the formation of the macrosegregation pattern in large steel ingots. Most of the characterization techniques use two-dimensional measurements. In this paper, a characterization method has been developed for equiaxed dendritic grains in industrial steel castings. A total of 383 contours were drawn two-dimensionally on twelve 6.6 cm^2^slices. A three-dimensional reconstruction method is performed to obtain 171 three-dimensional grains. Data regarding the size, shape and orientation of equiaxed grains is presented and thereby shows that equiaxed grains are centimeter-scale complex objects. They appear to be a poly-dispersed collection of non-isotropic objects possessing preferential orientations. In addition, the volumetric grain number density is $2.2\times 10^{7}$ grains/$m^{3}$, which compares to the $0.5\times 10^{7}$ grains/$m^{3}$ that can be obtained with estimation from 2D measurements. The $2.2\times 10^{7}$ grains/$m^{3}$ value is ten-times smaller than that previously used in the literature to simulate the macrosegregation profile in the same 6.2 ton ingot.

## 1. Introduction

Steel manufacturing requires more homogeneous and cleaner products in terms of inclusions, chemical segregations and porosity. Chemical segregations at the product scale cannot be reduced once the product is fully solidified and are responsible for the differences of mechanical properties in rolled products. Those macrosegregations arise because of the relative motion between the sedimenting solid grains and the thermosolutal convected liquid phase. Consequently, the number, shape and size of the grains are expected to have a great impact on the relative motion and thus on the final macrosegregation profile. Good characterization methods to measure grain properties are of primary importance. Moreover, improvements in casting processes need multi-scale and multi-physics numerical modeling. To be realistic, these models need an extensive description of the grain properties at the beginning of solidification [1,2,3,4,5,6,7,8,9,10]. Those numbers are not accessible for industrial castings, and postmortem techniques need to be developed.

Conventional metallography uses 2D measurements for equiaxed dendritic grain size measurements [11,12,13,14,15]. More recently, computer-aided methods that take binary images as the input can be found in the literature. On these binary images, combined segmentation/erosion methods to isolate individual dendrites have also been applied to equiaxed dendritic structures [11,12], as well as intercept techniques, [13,14,15]. They only measure primary and secondary dendrite arm spacings. Moreover, these image processing techniques give only qualitative and non-comparative results as they are very sensitive to the segmentation and filtering steps, which remove very small objects. Finally, the major drawback of these techniques is that for non-convex 3D objects, no stereological relationship exists to estimate the 3D properties of the grain distribution. It is then necessary to move to 3D in order to characterize equiaxed dendrites.

Advanced techniques like computed X-ray tomography [16,17,18,19] or serial cutting/polishing have been used to assess the real 3D grain size of dendritic structures [20,21]. The large size of equiaxed grain structures, which is in the order of magnitude of 1 , in addition to the low chemical contrast as encountered in heavy steel ingots makes using X-ray computed tomography quite impossible. The same problem exists with commercially available serial polishing microscopes for steel. For such large structures, conventional cutting remains the best solution. An example was given by Laren and Fredriksson in 1972 to identify the shape of one columnar grain (five slices) and one equiaxed grain (three slices) [22]. However, the small number of cutting planes and number of grains prevented drawing a general conclusion about dendritic equiaxed grains.

The current study in this paper proposes a macro serial cutting method to characterize 3D centimetric equiaxed grains and to estimate the 3D volumetric equiaxed grain density for industrial steel ingots. The method used along with its advantages and its drawbacks are presented with one example of an industrial ingot. It is depicted that the grain density, the characteristic size, the shape and the orientation of the 3D equiaxed grain distribution can be determined with this method.

## 2. Materials and Methods

### 2.1. Material and Microscopy

**Figure 1:**
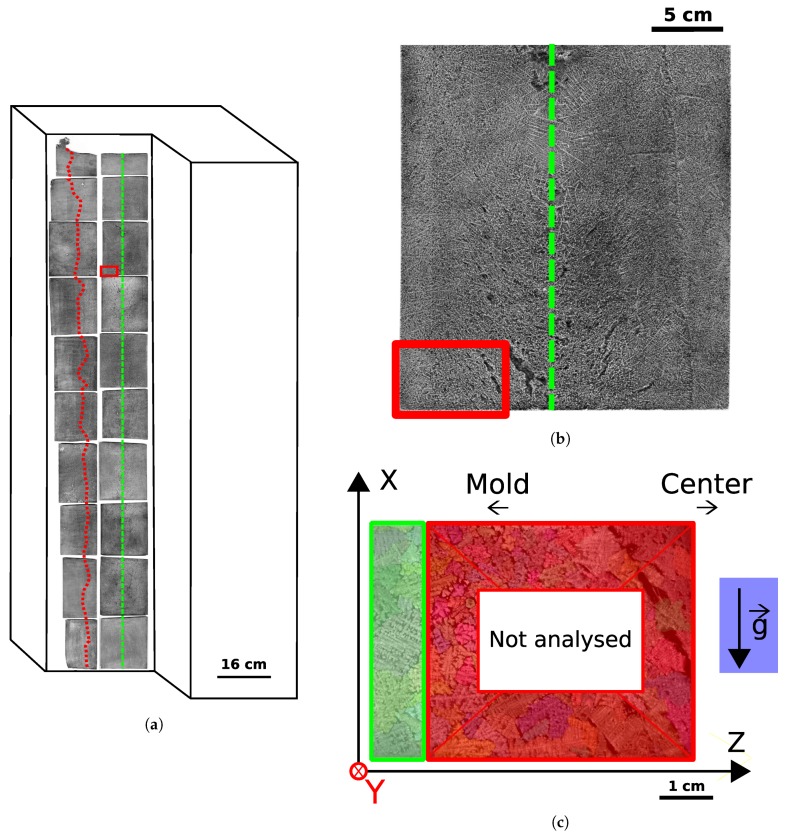
Description of the sample position in a 6.2 ton 100Cr6 ingot. The total height of the ingot is 267 cm. The red dotted line is the columnar-to-equiaxed transition. The green dashed line is the central axis of the ingot. (**a**) Macroetched central slice inside a 3D schematic view of the ingot. (**b**) Position of the sample for serial cutting. (**c**) Zoom on the red rectangle with the position of the serial cut metal in the green rectangle. The normal vector to the slices is directed along the Z axis.

The steel grade used in this study is 100Cr6 (ASTM 52100). 100Cr6 is a through hardening steel used for bearing steel. It is industrially cast as ingots, and its nominal composition is given in Table 1. A 6.2 ton 100Cr6 bottom poured ingot was cast in an Asco Industries plant at Fos-sur-Mer, France, for use in this study. as seen in Figure 1a.

As a part of conventional metallographic characterization, one central slice was cut along the main axis of the ingot and divided into thirty sections. Polishing followed by macroetching in a warm 15 wt % HCl aqueous solution was performed on each section to reveal the solidification structure. It must be noted that electron back-scattering diffraction cannot be used for characterization of low-alloyed steel primary structures. 100Cr6 fully solidifies in γ-austenite. At lower temperatures, primary austenite undergoes solid state phase transformations, and all the primary crystallographic structure is transformed. In the as-cast state, 100Cr6’s microstructure is mostly fully pearlitic. Dendrites exist at room temperature only as chemical segregations that can be revealed with an appropriate reagent.

Macrostructures were imaged on a high resolution Expression 12000XL EPSON scanner (EPSON, Suwa, Japan). Of these etched structures, one sample has been chosen in the equiaxed zone and outlined in Figure 1. The sample is located 60 cm from the top of the ingot and 1.5 cm from the central axis of the ingot (drawn as the green dotted line). The distance from the edge of the sample to the columnar-to-equiaxed transition (drawn as a red dashed line) is 7 cm. The top and the bottom of the ingot are located at the left and right side of the figure, respectively. The sample dimensions are 1.75 cm×3.8 cm×5.1 cm.

### 2.2. Serial Cutting

On the sample taken from the ingot in Figure 1a, 12 slices of 0.7 mm in thickness were cut by wire erosion, the diameter of which is 300 μm. Those slices are taken in the green part of Figure 1c with their normal vectors parallel to $\vec{z}$. The total analyzed thickness was 1 cm, which is the width of the green area along the ingot radial direction, $\vec{z}$. Marks were made before the cutting to ensure that each slice was properly tracked and the final slice had dimensions of 1.75 cm;×3.8 cm;×0.07 cm;. Optical microscopy was used to control the slice widths, where no defect in parallelism was observed.

### 2.3. Manual Outlining of Individual Grains

All the slices were ground up to 4000 SiC paper, and the final polishing was performed with a 3 μm diamond suspension. The solidification structure was revealed by warm etching in a supersaturated picric acid aqueous solution (Bechet–Beaujard reagent). Micrographs were taken with a Axioplan 2/Axiocam MRc5 optical microscope (Carl Zeiss, Oberkochen, Germany). The motorized platform combined with Axiovision software (version 4.6) allowed macroscopic tile images to be created.

The freeware Fiji was used for image analysis [23]. Manual identification of each 2D grain was performed after histogram equalization. Orientations of the dendrite primary axis and secondary arms were used to outline one grain on the first slice. Once a grain was outlined on its first slice of appearance, primary axis and secondary axis of the same grain were once again used as indicators for outlining on the other slices. The process was repeated until the grain could not be seen on the other slices or until the last slice was reached. Other grains were then outlined with the same procedure. A total of 390 2D contours were drawn manually.

**Figure 2:**
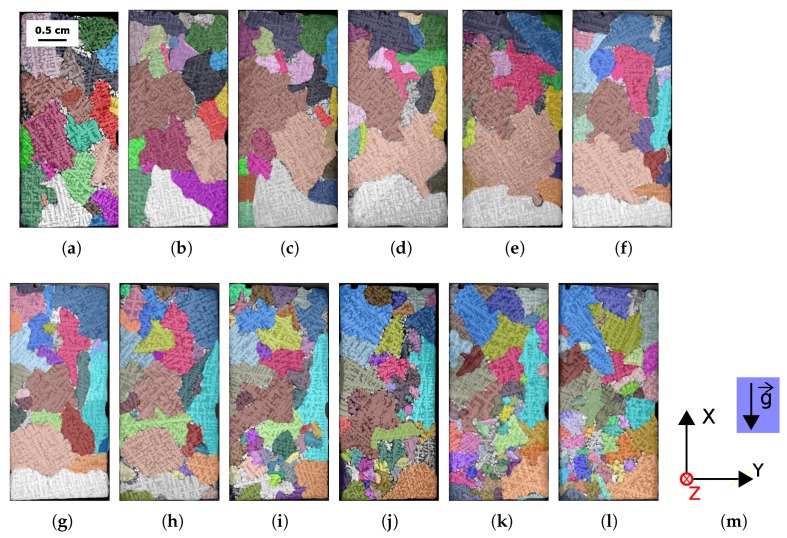
Solidification microstructure after FiJi processing. In the web version, each grain maintains the same color through all slices. (**a**) Z = 0 cm; (**b**) Z = 0.11 cm; (**c**) Z = 0.22 cm; (**d**) Z = 0.33 cm; (**e**) Z = 0.44 cm; (**f**) Z = 0.55 cm; (**g**) Z = 0.66 cm; (**h**) Z = 0.77 cm; (**i**) Z = 0.88 cm; (**j**) Z = 0.99 cm; (**k**) Z = 1.1 cm; (**l**) Z = 1.2 cm; (**m**) Associated coordinated system.

A custom Fiji macro was used to store all the data about each 3D grain as a Fiji region of interest. The macro also produced a colored image of each individual slice, where each grain remained the same color on each slice. The final output is presented in Figure 2.

### 2.4. 3D Reconstruction

**Figure 3:**
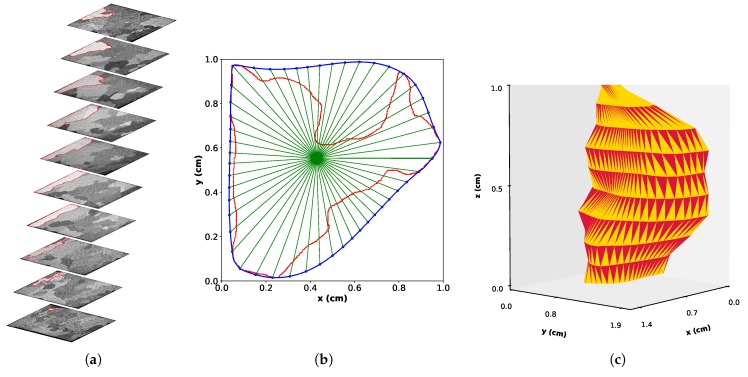
Principle of 3D reconstruction with 2D outlining followed by polar discretization. (**a**) Same grain outlined on each slice. (**b**) Concave contours (red) resulting from the 2D outlining in one slice of (**a**) with its convex Hull contour (blue). Green lines shows the polar discretization of the blue contour. (**c**) 3D reconstructed equiaxed grain. This the same grain as in (**a**). This grain is also colored in light grey at the bottom of each slice in Figure 2.

A program was written in Python 3 to reconstruct each grain in 3D as shown in Figure 3. After retrieving the data per grain from Fiji (as shown in Figure 3a) using the read-roi functions developed by Mary et al. [24], each 2D contour belonging to the same 3D grain needed to be discretized. As the main goal is surface reconstruction, polar discretization was done on each contour with the same predefined array of angles. Henceforth, each angle is represented as a line, which is depicted in Figure 3b. If random points were taken on two successive contours, vertices could cross each other, and the surface would not be properly reconstructed. The chosen grain in Figure 3 is the white grain at the bottom of each slice in Figure 2a–i.

To ensure that the solution is unique for each angle, the discretization must be performed on a convex surface. The convex hull of the contour, i.e., the external blue contour in Figure 3b, was used. Each angle was then computed with respect to the center of mass of the new convex contour. This point is the origin of all the blue lines, and each line represents one angle.

The intersection between the lines and the convex Hull contour are the points used for the reconstruction of the surface in 3D between two sets of contours. The final result for one grain is shown in Figure 3c. The number of 3D grains retrieved in the sample volume from the twelve slices is 171. This 3D reconstruction is only used for graphical purposes.

For the analysis, the use of grain area and grain volume is inconvenient in the international unit system. Image analysis studies often compare lengths by using equivalent circle and sphere diameters. Those equivalent diameters do not make any sense in the present case because the 2D dendrite morphology is very far from being circular.

**Figure 4:**
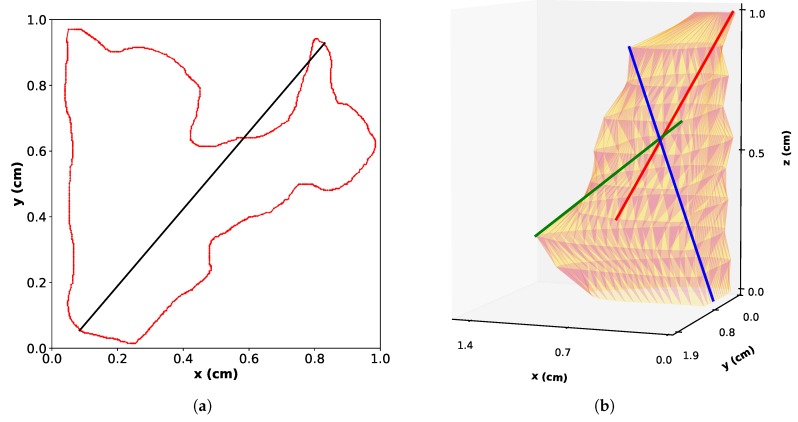
Principle of Feret calculation. (**a**) 2D Feret for the contour of Figure 3b. The 2D Feret diameter is drawn as the black line. (**b**) 3D principal, secondary and tertiary Feret for the grain of Figure 3c. The 3D principal, secondary and tertiary Feret are drawn as the red, green and blue lines, respectively.

The Feret diameter, which is defined as the longest distance between two points in a cloud of points, can be considered as a more physical length to describe the grains. For 2D objects, Feret diameters are found from the 2D point clouds created by the manually-drawn concave contours. An example of Feret estimation is shown in Figure 4a for the grain contour from Figure 3a. It can be seen that for concave contours, the Feret diameter may not lie in the drawn contour. For 3D objects, the point cloud for Feret calculation is the sum of all the 2D contours that belong to an individual grain.

In our dataset, it has been verified that grain area and grain volume are respectively proportional to the square and cube of the 2D and 3D Feret diameters. As a consequence, these two parameters are relevant to describe the geometry of equiaxed dendrites. They are also quite easy to handle, to compute elongation factors and also to define orientation angles. Feret diameters (2D or 3D) alone are sufficient to precisely describe a 3D object because they cannot describe if an object is isotropic, and other size parameters need to be introduced in other directions of the space.

Secondary and tertiary Feret diameters are defined similarly to 3D Feret diameter (also called 3D principal Ferret diameter): the secondary Feret diameter is defined as the longest distance found in a direction perpendicular to the principal Feret diameter, whereas the tertiary Feret diameter is the longest distance perpendicular to the principal Feret diameter and the secondary Feret diameters. To ensure that those other Feret diameters always numerically exist, the perpendicularity condition needs to be relaxed with a tolerance of ± 5 ${}^{\circ }$. This is because of the lack of data in between the different slices that creates non-existing z positions and angles in the 3D cloud point. Examples of the 3D principal, secondary and tertiary Feret diameters are given on Figure 4b for the 3D grain of Figure 3c. It has to be noted that with those definitions, nothing ensures that the three 3D Feret cross all at the same unique point.

## 3. Results

### 3.1. 2D Identification of 3D Grains

**Figure 5:**
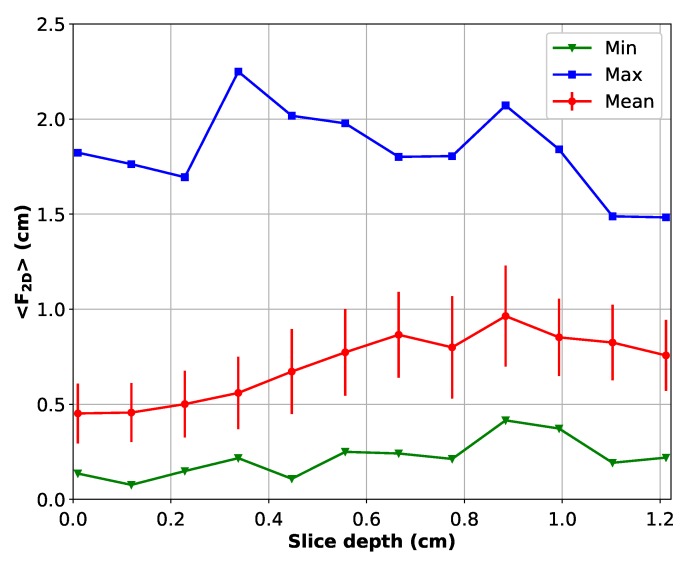
Evolution of the mean 2D Feret diameter on all the slices.

As said in Section 1, common metallographic measurements are two dimensional. With our dataset, it is possible to retrieve this kind of measurement using only the 2D contours. If each slice were considered individually, the average 2D Feret diameters, $<F_{2D}>$, would vary through the slices, as shown in Figure 5. The represented error bars are 50 % of the standard deviation (0.5·σ). The mean value, of all the grains on all slices, is 0.67 cm with a standard deviation of 0.15 cm. This mean value is not consistent with the variation observed through all the slices. On the first slice, the mean 2D Feret is 0.45 cm, whereas on the last seven slices, the mean 2D Feret length is close to 0.85 cm±0.1 cm. In other words, it means that grain size measurements can give results varying up to a factor of two just by changing the sampling position for the 2D measurement by 1 cm, i.e., measuring on the 0 cm slice depth or measuring on the 1 cm slice depth. It is the proof that gradients of structure sizes exist on very short scales in industrial ingots.

**Figure 6:**
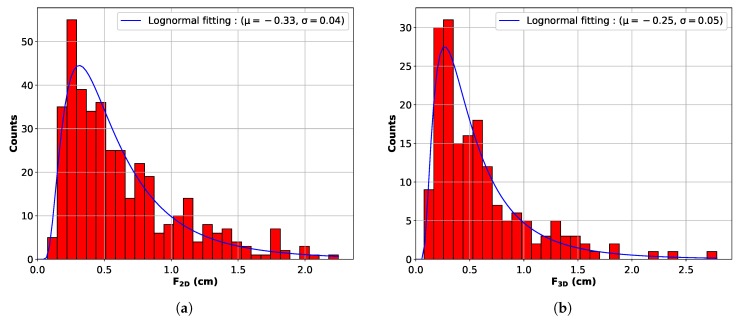
Histogram of measured Feret. (**a**) 2D Feret diameters. (**b**) 3D Feret diameters.

Figure 6a shows the distribution in terms of 2D Feret diameter (F${}_{2D}$). The distribution follows a classical log-normal law that has been fitted with the blue line. The low probability of cutting 3D grains in a plan containing the principal Feret diameter is well described by a log-normal distribution law. The principal mode and the maximum Feret diameter are 0.72 cm and 2.2 cm, respectively. However, there is no information regarding the real shape of the grain. It is therefore impossible to understand what really is the grain size and also, for example, to infer if the grain distribution is uniform, bimodal or even more complex.

The volumetric grain number density can be at first approximated by dividing the surface grain number density by the mean 2D Feret diameter for each slice [25]. Although it is only valid for monodisperse and convex objects, it is a fast way of providing a lower boundary for the real 3D value. With this workflow, values from $0.5\times 10^{7}$ – $2\times 10^{7}$ /$m^{3}$ are retrieved. Those values are strongly under-evaluated because the calculation performed averages the values of the Feret diameter on each slice.

### 3.2. 3D Grain Structures

**Figure 7:**
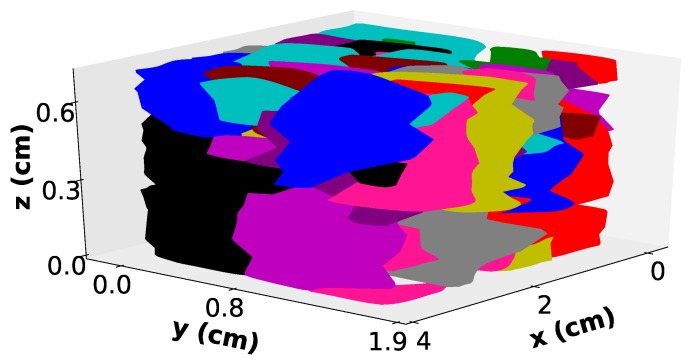
3D reconstructed envelope of the dendritic microstructure. Each color in the web version accounts for one grain.

The final 3D result is thereby presented in Figure 7. Each reconstructed grain envelope was given a random color. As for 2D measurements, it is also possible to observe the distribution of 3D Feret diameters as shown in Figure 6b. Consequently, the number of 3D Feret diameters is much lower than the number of 2D contours and equals the number of grains, i.e., 171 grains. The shape of the distribution is the same, but the characteristic values are slightly different. The principal mode and maximum values are respectively 0.8 cm and 2.75 cm. The ratio between 2D and 3D mean Feret diameters is 0.9. A short Monte Carlo study on 1000 random slices on monodisperse cubes and spheres gives values of 0.7 and 0.94, respectively, for the same ratio. The volumetric grain number density is $2.2\times 10^{7}\mathrm{grains}/m^{3}$.

### 3.3. 3D Grain Properties

**Figure 8:**
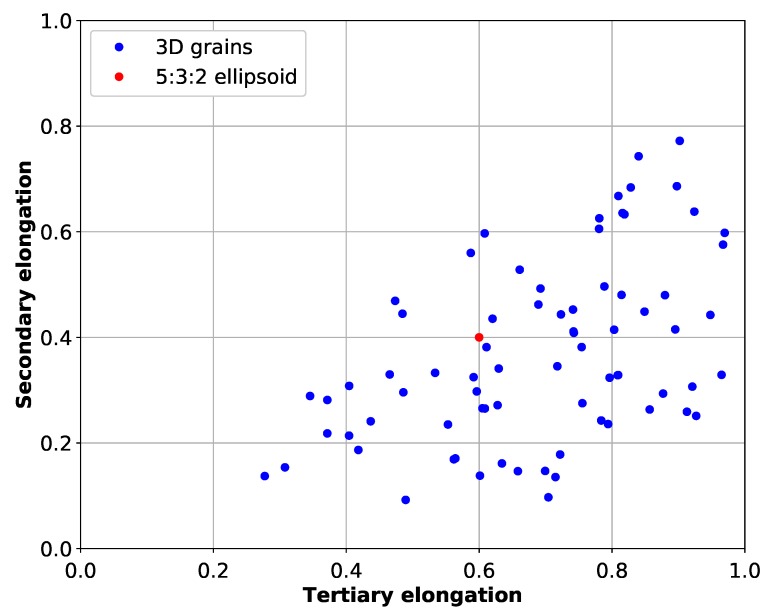
Comparisons of secondary and tertiary equiaxed dendritic grain elongation factors.

In 3D, the Feret diameter is only a partial indicator of grain size. Further insight can be gained by finding the longest size in directions perpendicular to the principal Feret diameter. After calculations, three Feret diameters (principal, secondary and tertiary) are obtained in the direction perpendicular to each other with a decrease in lengths. For isotropic objects like spheres, the secondary Feret diameter equals the principal Feret diameter. For non-isotropic objects, they provide a quick way to determine if the objects are elongated. Elongation factors, represented by secondary or tertiary Feret diameters divided by the principal Feret diameter values, are plotted in Figure 8. From the two elongation factors, it can be seen that the dendritic grains are non-isotropic objects with complex shapes. The mean elongation factors are equivalent to a 5:3:2 ellipsoid. The most symmetrical case that exists is a 5:5:4 ellipsoid, and the most elongated one is equivalent to a 10:5:2 ellipsoid. There is no isotropic case in the analyzed volume. In comparison, the 2D elongation mean case is a 5:3 ellipse. The 2D value is close in comparison to the 3D one, but does not give any information regarding the 3D shape.

**Figure 9:**
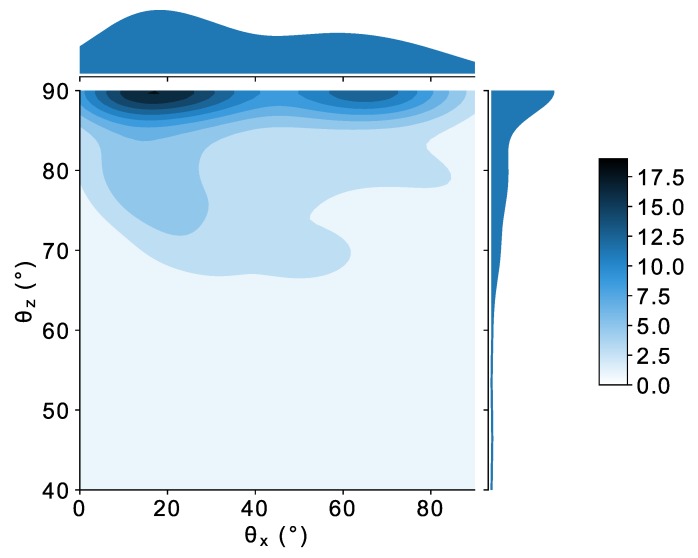
Density map for 3D principal Feret orientations with respect to the x and z directions. Density function shape for $\theta_{x}$ and $\theta_{z}$ are respectively plotted parallel to the corresponding axis. The *z* direction is a radial direction of the ingot, and the *x* direction is opposite gravity.

The orientation of dendritic grains is also of interest. The orientation of the principal Feret diameter is represented in Figure 9. To obtain a density plot, kernel density estimation was applied to the data with the use of the seaborn python library, [26]. This library is very useful because it allows one to directly see the density function for each variable along with the two-dimensional estimated density function. The colorscale values are directly proportional to the number of points by unit of area in the 2D space ($\theta_{x}$, $\theta_{z}$). It can be seen that most of the principal Feret diameters are perpendicular to the slicing direction, $\vec{z}$, which is also the radial direction of the ingot. Moreover, the distribution of the principal Feret diameter orientation has two main characteristic orientations with respect to the *x* direction ($\vec{x}$ is oriented along $-\vec{g}$, which is the opposite direction of gravity). Those characteristic values are located at the center of the darker spots in Figure 9 at 10 ${}^{\circ }$ and 65 ${}^{\circ }$ with respect to $\vec{x}$. Those characteristic orientations do not appear to be related in any way to the principal Feret diameters.

## 4. Discussion

The main drawback of the method is that all the grains are kept during the analysis. Usually in image analysis, objects that intercept picture sides are removed [27]. This is not the case in the present analysis because of the small size of each slice and also the large grain size. It is a source of errors in the estimation of grain size, orientation and shape factors. However, it can be corrected in the evaluation of the volumetric grain number density, which is the most important parameter in this study. This can be done by adding factors regarding the number of picture sides that the grain is crossing [27]. In the same way, some grains are only observed on one slice. The size of these grains is only estimated in 2D and thus under-evaluated. Last, but not the least, grains finer than the total distance between two observation planes are lost.

As previously stated, common practice for equiaxed grain size determination is 2D measurement, which is a fast way to obtain rudimentary results. In this case, the 2D grain size measurements allow us to establish a mean grain size of 0.67 cm. Previous measurements reported by Kumar et al. on the same kind of ingots gave experimental grain size values on the order of 0.1 cm via the ASTM E-112 intercepts technique [28]. This kind of measurement gives values related to the secondary dendrite arm spacing and is by nature not adapted to dendritic equiaxed grain size measurements. Secondary arms that intercept the measurement line will be counted as the intercept and will decrease the mean intercepted length. The norm would only be applicable to the manually outlined grain contours that will be 2D convex contours. This case would be equivalent to the usual measurements of austenitic grain size.

In this study, 3D measurements show that grain size heterogeneities can arise in an industrial ingot on a scale less than 1 cm. On the first slice, the mean 2D Feret is 0.45 cm, whereas on the last seven slices, the mean 2D Feret length is close to 0.85 cm±0.1 cm. The 25 % of the largest grains fill 80 % of the analyzed volume. Equiaxed grains appear to be a polydisperse collection of objects, which was not possible to prove with two-dimension analysis alone. The ratio of 0.94 between 2D and 3D mean Feret diameters is a direct consequence of the polydispersity. Random cuttings of each 3D dendrite alone would not give results as close to unity because of the low shape isotropy (i.e., secondary and tertiary elongation factors far from one).

Three-dimensional analysis indicated that grains are elongated and not fully isotropic. The real shape of dendritic grains can be estimated by ellipsoids at the first order. This means that no good estimation, in terms of volumetric grain number density, can be made with only 2D measurements. For example, by neglecting the concave shape of equiaxed dendrites, it is possible to estimate the volumetric grain number density by dividing the mean 2D surface grain number density by the 2D mean Feret diameter, which is $0.7\times 10^{7}$ /$m^{3}$. Though the order of magnitude is correct, the numerical value is four-times lower than the 3D value. It has to be noted that 2D and 3D Feret diameter calculations are performed on concave contours. The 2D and 3D Feret diameters as defined in this study may not be fully contained respectively in the surface or volume of interest. This underestimation accounts for one part of the difference between the volumetric density estimated from 2D measurement and the value estimated from the number of 3D grains.

This parameter is crucial to validate macrosegregation numerical models currently under development like finite volume or finite element models [4,29]. Numerical predictions by Kumar et al. for the ingot investigated in this study, consider three different cases with varying initial volumetric densities: $1\times 10^{8}$ /$m^{3}$, $5\times 10^{9}$ /$m^{3}$ and $5\times 10^{10}$ /$m^{3}$, respectively [1]. According to the authors, those initial values produce final castings with structures that are fully-dendritic, mixed globular/dendritic and fully-globular, respectively. The experimental value, $2.2\times 10^{7}$ /$m^{3}$, is one order of magnitude less than the smallest value chosen for the simulation. The dendritic morphology as shown in Figure 2 clearly demonstrates that there is a good agreement between the experimental and the predicted morphology.

Finally, equiaxed grains are not randomly oriented. Their Feret diameters are always very close to the slicing planes. The most probable angle is perpendicular to the radial direction (relatively to the ingot) and less than 15 ${}^{\circ }$ disoriented from the direction of gravity. Another characteristic angle exists, perpendicular to the slicing direction, at an angle of 65 degrees from the direction of gravity. These two characteristic angles probably come from the settling period in the liquid prior to packing and from the shape of the liquid pool. This kind of textural effect cannot be retrieved from 2D characterization.

## 5. Conclusions

A method for 3D characterization in industrial castings with large equiaxed dendritic structures has been proposed. Slicing of one sample was performed by wire erosion, and outlines of each grain were made precisely. This manual method allows the accurate determination of 2D properties along with 3D estimation of grain size. Equiaxed grains appear to be complex, non-isotropic and non-randomly-oriented objects. The volumetric grain number density as calculated is close to $2.2\times 10^{7}$ grains/$m^{3}$. This value is ten-times smaller than the currently used value in the literature so as to simulate this kind of ingot. Numerical calculations need to be performed to identify the underlying cause of the determined textural effect. As a part of future work, new measurements should be carried out at different heights on the central line of an industrial ingot to see the evolution in terms of equiaxed structures. The number of slices has also to be increased to improve the precision of the technique. Such data could really help to understand what is the origin of equiaxed grains and to provide better inputs for the numerical models.

## Figures and Tables

**Table:** 

[]

**Table:** 

[]
[]

**Table 1:** Composition of 100Cr6 steel (wt %).

Grade	C	Mn	Si	Cr	Cu	Ni
100Cr6	0.98	0.32	0.22	1.4	0.12	0.14
